# Strong spin-orbit coupling inducing Autler-Townes effect in lead halide perovskite nanocrystals

**DOI:** 10.1038/s41467-021-23291-w

**Published:** 2021-05-21

**Authors:** Go Yumoto, Hideki Hirori, Fumiya Sekiguchi, Ryota Sato, Masaki Saruyama, Toshiharu Teranishi, Yoshihiko Kanemitsu

**Affiliations:** Institute for Chemical Research, Kyoto University, Uji, Kyoto Japan

**Keywords:** Semiconductors, Nonlinear optics

## Abstract

Manipulation of excitons via coherent light-matter interaction is a promising approach for quantum state engineering and ultrafast optical modulation. Various excitation pathways in the excitonic multilevel systems provide controllability more efficient than that in the two-level system. However, these control schemes have been restricted to limited control-light wavelengths and cryogenic temperatures. Here, we report that lead halide perovskites can lift these restrictions owing to their multiband structure induced by strong spin-orbit coupling. Using CsPbBr_3_ perovskite nanocrystals, we observe an anomalous enhancement of the exciton energy shift at room temperature with increasing control-light wavelength from the visible to near-infrared region. The enhancement occurs because the interconduction band transitions between spin-orbit split states have large dipole moments and induce a crossover from the two-level optical Stark effect to the three-level Autler-Townes effect. Our finding establishes a basis for efficient coherent optical manipulation of excitons utilizing energy states with large spin-orbit splitting.

## Abstract (web summary)

Here, Yumoto et al. demonstrate that for a halide perovskite with large spin-orbit splitting the optical Stark effect can give way to a three level Autler-Townes effect in the near-infrared region. The multiband nature of the effect potentially allows for further optical control over quantum states.

## Introduction

Hybridization between the quantum states and photon-dressed states induces the energy shifts of the quantum states. Such a light-driven coherent modification of the energy level spectrum is referred to as the optical Stark effect (OSE)^1^. The OSE provides a promising method for quantum state engineering and coherent optical modulation and has been intensively studied in excitonic two-level systems^2,3^, where the optical Stark shift is described by^3,4^

1 $$\delta {E}_{{\rm{Stark}}}\,=-{\it{\Delta}} +{\it{\Delta}}\sqrt{1+(\hslash {\varOmega }_{\rm{R}}/{\it{\Delta}})^2}$$

Here, detuning energy *Δ* is defined as *E*_0_ − $\hslash$*ω* (*E*_0_ is the exciton energy and $\hslash$*ω* is the photon energy) and $\hslash$*Ω*_R_ = *μE* is the Rabi frequency (*μ* is the transition dipole moment and *E* is the electric field of the light). As seen from Eq. (1), *δE*_Stark_ becomes larger with smaller *Δ* and larger $\hslash$*Ω*_R_. However, to minimize the real excitation effects which obscure the OSE, the driving field is restricted to large detuning energy and weak intensity, which limits the coherent controllability of exciton energy in the two-level systems^5^. On the other hand, the various excitation pathways in the multilevel systems enable more efficient and tunable optical manipulation of band-edge excitons through unique phenomena such as quantum interference^6,7^ and the Autler–Townes effect (ATE)^7–14^. In the multilevel systems realized in semiconductor nanostructures, the ground-to-exciton transition is modulated by coherently exciting the other ground-to-exciton transition separated by the fine-structure splitting^10^, the exciton–biexciton^6,7,11,12,15^, the intraexciton^13,14^, or intersubband transitions^9^. However, the multilevel system consisting of fine-structure split excitons and the exciton–biexciton system are realized only at low temperatures because the fine-structure splitting and biexciton binding energies are on the order of several tens of μeV and less than several tens of meV, respectively. In addition, such small differences between the ground-to-exciton transition and the other transitions limit the control-light energies to those close to the exciton energy, where near-infrared control light with wavelengths shorter than 1 μm has been used^6,7,10,12,15^. On the other hand, since the intraexciton and intersubband transition energies lie in the range of a few meV to less than a few hundred meV, terahertz to mid-infrared light is required to modulate the ground-to-exciton transition in the multilevel systems based on these transitions^9,13,14^. Therefore, the implementations of multilevel systems for coherent optical control and modulation are restricted to limited control-light wavelength ranges shorter than 1 μm or longer than mid-infrared wavelengths and to cryogenic temperatures.

Lead halide perovskites (LHPs) are promising materials for photonic and optoelectronic devices with low-cost solution processability and high photoluminescence quantum yields. They are also attractive platforms for studying strong light-matter interaction. Indeed, large OSE has recently been observed at room temperature under the pump excitation with photon energies >1.55 eV^4,16–18^. Under such excitation conditions, the OSE in LHPs can be described by two independent excitonic two-level systems, reflecting the twofold degeneracy of the valence and conduction band (CB) edges. Owing to the presence of the heavy metal atom in LHPs, a relativistic coupling between electronic spin and orbital momentum, i.e., spin–orbit coupling, becomes pronounced. Thus, in cubic phase LHPs, the strong spin–orbit coupling splits the CB with an overall *p* symmetry into a band-edge split-off state and higher-energy CB states^19–21^. The spin–orbit splitting energies of LHPs are reported to be >~0.7 eV^19,21–26^, which are large compared with those of the benchmark semiconductor GaAs (0.34 eV)^27^ and monolayer transition metal dichalcogenides (up to 0.42 eV)^28^. Then, if the inter-CB transitions can be coherently excited, we can expect the multilevel scheme to be implemented in LHPs and provide an opportunity to effectively modulate the band-edge transitions using the near-infrared control light with wavelengths longer than 1 μm. From **k·p** theory^29^, although the CB states share the same overall *p* symmetry, the inter-CB transitions are allowed because the **k·p** interaction causes more mixing of the *p*-like and the *s*-like Bloch states with an increasing deviation of the wave vector from the R point. However, in spite of its potential for providing a unique coherent control approach, coherent optical manipulation utilizing spin–orbit split states has never been studied and it is totally unknown how the inter-CB transitions affect the light–matter interaction.

Here, we report the observation of an anomalous enhancement of the exciton energy shift in CsPbBr_3_ perovskite nanocrystals (NCs) at room temperature as the pump energy is widely changed from the visible to near-infrared wavelength region. It is found that the dramatic change of the energy shift reflects a crossover from the two-level OSE to the three-level ATE. We show that this unique coherent manipulation is realized because the inter-CB transitions between spin–orbit split states have a large dipole moment of 25 D. In addition, the pump-induced exciton population, which impedes ultrafast optical responses^30^, is suppressed in the three-level ATE region. This suggests efficient pathways to realize ultrafast optical switching. Our finding provides a novel coherent optical modulation scheme based on strong spin–orbit coupling, which extends control-light wavelength range to near-infrared telecommunication wavelength and operation temperature to room temperature.

## Results

### Optical transitions in CsPbBr_3_ NCs

**Fig. 1:**
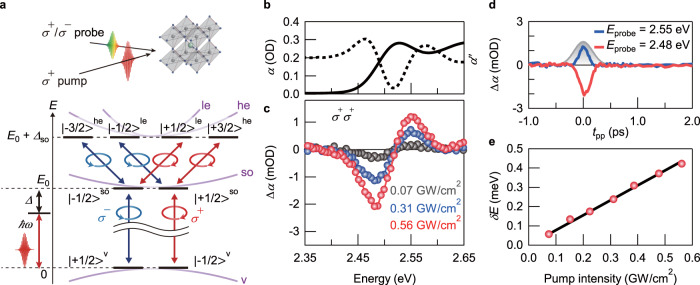
Optical transitions and two-level OSE for *Δ* = 0.20 eV in CsPbBr_3_ NCs. **a** Illustration of the circularly polarized pump–probe configuration (top) and the cubic CsPbBr_3_ band structure around the R point (bottom). The light electron, heavy electron, and split-off CBs, and valence band are denoted by le, he, so, and v, respectively. The transition selection rules between band-edge states and between CB states are shown. The detuning energy *Δ* is defined as the difference between the pump energy $\hslash$*ω* and the band-edge exciton transition energy *E*_0_. **b** Absorption spectrum of the CsPbBr_3_ NCs (solid curve, left axis) and its second derivative (dashed curve, right axis). **c** Change in the absorption spectra obtained at *t*_pp_ = 0 ps in the *σ*^+^*σ*^+^ configuration for three different pump intensities. The pump is red-detuned from the band-edge exciton peak by 0.20 eV. **d** Transient absorption dynamics probed at 2.48 and 2.55 eV for a pump intensity of 0.56 GW/cm^2^. The gray-shaded region shows the cross-correlation between the pump and probe pulses. **e** Pump intensity dependence of the estimated energy shift of the band-edge exciton. The black line shows the fitting result.

In Fig. 1a, the bulk band structure of CsPbBr_3_ in the cubic phase is illustrated. The band edges are located at the R point in the Brillouin zone. While the *s*-like valence band consists of Pb 6*s* and Br 4*p* orbitals, the CB has an overall *p* symmetry arising mainly from Pb 6*p* orbitals^19^. A strong spin–orbit coupling further splits the CB by energy spacing of *Δ*_so_ into a band-edge split-off state with a total angular momentum *J* of 1/2 (|±1/2>^so^) and higher-energy CB states with *J* = 3/2, heavy (|±3/2>^he^) and light (|±1/2>^le^) electron states^19–21^. Here, |*m*> denotes the state with the total electron angular momentum projection of *m*. The band-edge optical responses of CsPbBr_3_ NCs, which have a cubic crystal structure^22^, are determined by the transitions from valence band-edge states |±1/2 >^v^ to split-off CB states |±1/2>^so^. The transition from |−1/2 >^v^ (|+1/2 >^v^) to |+1/2>^so^ (|−1/2>^so^) occurs with *σ*^+^ (*σ*^−^) light, where *σ*^+^ (*σ*^−^) denotes the right (left)-handed circular polarization. In the excitonic picture, the transition corresponds to the *σ*^+^ (*σ*^−^)-light excitation of a band-edge exciton with the total exciton angular momentum of *J*_ex_ = +1 (−1). The pump-induced energy shift of the exciton with *J*_ex_ = +1 (−1) is probed by *σ*^+^ (*σ*^−^) light in the pump–probe measurements. In addition to the band-edge optical responses, the transitions within the CB states are also possible at the finite deviation of the wave vector from the R point and provide another route to manipulate the band-edge states. From angular momentum conservation, the transitions from |+1/2>^so^ to |+3/2>^he^ (|−1/2>^le^) and from |−1/2>^so^ to |+1/2>^le^ (|−3/2>^he^) are allowed by *σ*^+^ (*σ*^−^) light (Fig. 1a).

### Two-level optical Stark effect

To investigate coherent modulation of band-edge exciton transitions, we performed pump–probe spectroscopy on hexane-dispersed CsPbBr_3_ NCs at room temperature with *σ*^+^ monochromatic pump pulses and *σ*^+^ or *σ*^−^ white light probe pulses (Fig. 1a). The background signal from the hexane was removed by measuring the transient absorption of the hexane reference sample (Supplementary Fig. S2). Our samples were synthesized by a hot-injection method (see “Methods”) and had an average NC size of 6.9 nm. According to the exciton Bohr diameter of 7 nm for CsPbBr_3_^22^, they lie in the intermediate confinement regime. Figure 1b shows the steady-state absorption spectrum of CsPbBr_3_ NCs and its second derivative. By assuming an absorption band with a Gaussian profile^31^, the fit of the second derivative results in a band-edge exciton transition energy *E*_0_ of 2.519 eV and a linewidth *Γ* of 0.079 eV (full width at half maximum). The change in the absorption spectra Δ*α* induced by *σ*^+^ pump was measured by *σ*^+^ probe (*σ*^+^*σ*^+^ configuration), where the pump was red-detuned from *E*_0_ by 0.20 eV (Fig. 1c). At all pump intensities, we observed the dispersive-shaped Δ*α* which indicates the pump-induced blueshift of *E*_0_. From Δ*α* as a function of the pump–probe delay *t*_pp_ at pump intensity *I*_pump_ of 0.56 GW/cm^2^ (Fig. 1d), it is shown that the energy shift occurs only during the pump excitation, indicating that the observed blueshift arises from the OSE. In Fig. 1e, we show the *I*_pump_ dependence of the energy shift *δE* estimated by the spectral weight transfer method (see Supplementary Text S1). By using Eq. (1) including local field correction (see Supplementary Text S2), we fitted the data in Fig. 1e and obtained the transition dipole moment of the band-edge exciton *μ* = 19 D, which is about three times larger than the reported value in GaAs quantum wells^2^.

### Crossover from the two-level optical Stark effect to the three-level Autler–Townes effect

**Fig. 2:**
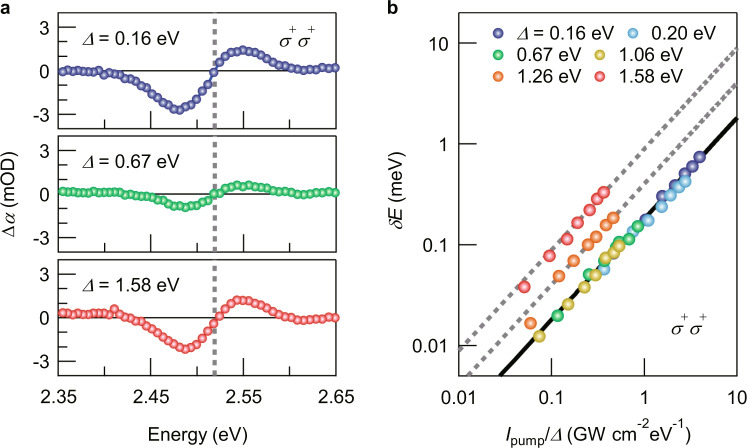
*Δ* dependence of the energy shift in the *σ*^+^*σ*^+^ configuration. **a** Δ*α* spectra at *t*_pp_ = 0 ps for different detuning energies. The pump intensity was set to 0.56 GW/cm^2^. The gray dashed line indicates the band-edge exciton transition energy derived in Fig. 1b. **b** Log–log plot of the energy shift as a function of *I*_pump_/*Δ* for different detuning energies *Δ*. The black line shows the *δE*_Stark_ calculated using Eq. (1) for *μ* = 19 D. The gray dashed lines serve as guides to the eye.

Having confirmed the two-level OSE for *Δ* = 0.20 eV, we measured *δE* for different *Δ* in the *σ*^+^*σ*^+^ configuration. Figure 2a shows Δ*α* under the pump excitation with *I*_pump_ = 0.56 GW/cm^2^ for *Δ* = 0.16, 0.67, and 1.58 eV. We chose *Δ* as *Δ* > *Γ* to prevent direct excitation. However, we still observed an incoherent component with a slow decay in the dynamics of Δ*α* for *Δ* = 0.16 eV (see Supplementary Text S3). Thus, the Δ*α* for *Δ* = 0.16 eV is that obtained after subtraction of the incoherent component. As expected for the two-level OSE, Δ*α* reduces when *Δ* is increased from 0.16 to 0.67 eV. In stark contrast to this normal OSE, it is observed that Δ*α* starts to increase again with *Δ* further increasing from 0.67 eV. To take a closer look at this non-monotonic behavior, we plot *δE* as a function of the *Δ*-normalized *I*_pump_ for various *Δ* in Fig. 2b (see also Supplementary Fig. S4). We can see that the plots for *Δ* ≤ 1.06 eV lie on a single line. This behavior can be explained by considering that Eq. (1) is approximated to ($\hslash$*Ω*_R_)^2^/(2*Δ*) when *Δ* ≫ $\hslash$*Ω*_R_, which our measurement conditions ($\hslash$*Ω*_R_ = 14.2 meV at *I*_pump_ = 0.56 GW/cm^2^) fulfill. This is clearly seen from the good agreement between the experimental results for *Δ* ≤ 1.06 eV and *δE*_Stark_ calculated with *μ* = 19 D. On the other hand, for *Δ* > 1.06 eV, while also showing linear dependence on *I*_pump_/*Δ*, the *δE* deviates from those expected from the two-level OSE.

**Fig. 3:**
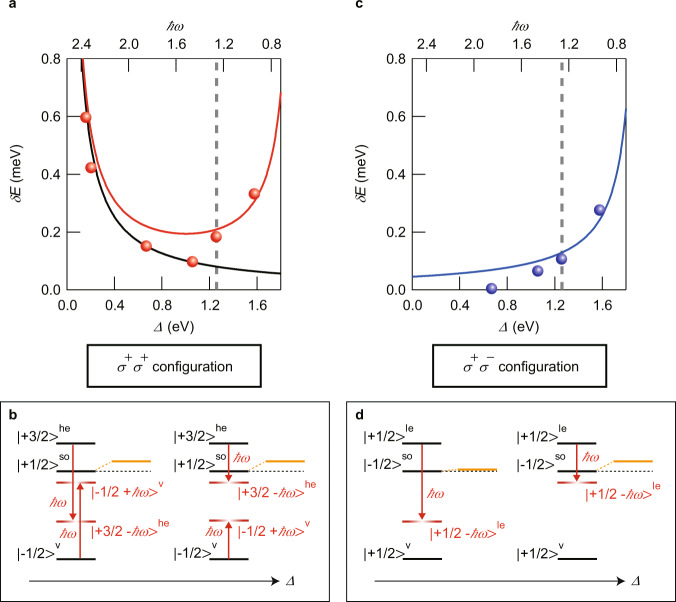
Crossover from two-level OSE to ATE. **a** *Δ* dependence of the energy shift in the *σ*^+^*σ*^+^ configuration for a pump intensity of 0.56 GW/cm^2^. The red curve shows the calculated energy shift based on the three-level system model. The black curve is the *δE*_Stark_ calculated using Eq. (1) for *μ* = 19 D at the corresponding pump intensity. The top scale shows the pump energy. The gray dashed line indicates half of the band-edge exciton energy. **b** The energy diagrams of the *Δ*-dependent hybridization of the photon-dressed states and the lowest CB state |+1/2>^so^, which can be probed in the *σ*^+^*σ*^+^ configuration. The orange lines represent the eigenstates after the hybridization with the photon-dressed states. The hybridizations of the other photon-dressed states with |−1/2 >^v^ and |+3/2>^he^ are not shown, for simplicity. **c**, **d** *Δ* dependence of the energy shift (**c**) and the corresponding energy level diagram (**d**) in the *σ*^+^*σ*^−^ configuration.

In order to reveal the mechanism of the observed anomalous energy shift, we plot the *Δ*-dependence of the *δE* observed for *I*_pump_ = 0.56 GW/cm^2^ in the *σ*^+^*σ*^+^ configuration (Fig. 3a). As *Δ* is increased, the *δE* starts to increasingly deviate from *δE*_Stark_ calculated with *μ* = 19 D, exhibiting ~5-fold enhancement compared with the estimated *δE*_Stark_ at *Δ* = 1.58 eV. The dramatic increase of the *δE* in the near-infrared region indicates that as the pump energy $\hslash$*ω* decreases, the excitation energy approaches some transition resonance below ~1 eV. Because the spin–orbit splitting energies in LHPs are reported to lie in the range of ~1 eV^19,21–26^ and the energy shift enhancement is independent of the pump polarization as described below, the resonant structure is considered to be related to the inter-CB transition. To identify the inter-CB transition, we performed pump–probe experiments under resonant excitation of the band-edge exciton transitions and estimated the energy spacing *Δ*_so_ of the inter-CB transitions to be 0.58 eV (see Supplementary Text S4). The estimated value of *Δ*_so_ is consistent with the experimentally reported value of the spin–orbit splitting energy of 0.8 eV in MAPbBr_3_ single crystals^26^. Note that size confinement has little effect on *Δ*_so_ because the confinement energy is estimated to be much smaller than *Δ*_so_ (see Supplementary Text S5). Thus, the slightly smaller value in NCs compared to 0.8 eV presumably stems from the reduced spin–orbit coupling due to structural distortions^32^. Thus, we consider a three-level system containing |−1/2 >^v^, |+1/2 >^so^, and |+3/2 >^he^ as shown in Fig. 3b. To extract the contribution of the inter-CB transition, we introduced the residual energy shift, defined as the energy shift after subtraction of *δE*_Stark_ from the measured *δE* (see Supplementary Text S6). From the fitting to the *I*_pump_ dependence of the residual energy shift for *Δ* = 1.26 and 1.58 eV, the transition dipole moment *μ*‘ of the inter-CB transitions was estimated to be *μ*‘ = 25 D. The large value of *μ*‘ larger than that of the band-edge transitions reflects the smaller energy spacing for inter-CB transitions compared with the band-edge transitions because the magnitude of the dipole moment is inversely proportional to the transition energy^33^. In addition, such a large dipole moment indicates the significant contribution of wave functions other than at the R point to the coherent nonlinear processes. This is consistent with the fact that the effects of non-zone-center wave functions having finite wave vectors are required to well describe two-photon absorption in zinc blende semiconductors^34^. Furthermore, it has recently been reported that inter-CB transitions are optically allowed and contribute to two-photon absorption spectra in MAPbBr_3_ single crystals^26^ and MAPbI_3_ thin films^24^.

To explain the whole trend observed in Fig. 3a, we modeled the three-level system by using the dressed state picture under rotating-wave approximation. Here, we neglect the counter-rotating term, which results in the Bloch–Siegert (BS) shift. The BS shift is also expected to give an enhancement of the energy shift with decreasing pump energy. However, the BS shift does not occur in the *σ*^+^*σ*^+^ configuration because it is forbidden by the transition selection rules in angular momentum for the counter-rotating field of the *σ*^+^ pump^35^. By diagonalizing the effective Hamiltonian, we obtained *δE* from the calculated energy shifts of |+1/2 >^so^ and |−1/2 >^v^ under the pump excitation (see Supplementary Text S7). The calculated *δE* is displayed as the red curve in Fig. 3a, and it reproduces the experimentally observed behavior of the energy shift. In addition, the good agreement between the black and red curves in Fig. 3a points to a negligible contribution of the inter-CB transition to the energy shift for *Δ* ≤ 0.20 eV. In fact, the value of *μ* estimated from the fitting involving the contribution of the inter-CB transition results in only a 4 and 6% reduction compared with the values obtained by using Eq. (1) for *Δ* = 0.16 and 0.20 eV, respectively. The enhancement in *δE* for larger *Δ* originates from the energy shift of |+1/2>^so^. For small *Δ*, the energy shift of |+1/2>^so^ arises from hybridization with the photon-dressed state |−1/2 + $\hslash$*ω* >^v^. On the other hand, as *Δ* increases and $\hslash$*ω* becomes close to the *Δ*_so_, the hybridization with |+3/2 − $\hslash$*ω* >^he^ starts to dominate the energy shift of |+1/2 >^so^, as depicted in Fig. 3b. In contrast to the energy shift of |+1/2 >^so^, the energy shift of |−1/2 >^v^ monotonically decreases with increasing *Δ* because |−1/2 >^v^ hybridizes with only the photon-dressed state |+1/2−$\hslash$*ω* >^so^. Such a crossover from the two-level OSE to the three-level ATE in the visible to the near-infrared region is the manifestation of the fact that the transitions between the spin–orbit split states in CsPbBr_3_ NCs have a large transition dipole moment.

To further confirm this interpretation, we measured *δE* by using the *σ*^−^ probe (*σ*^+^*σ*^−^ configuration). In this case, the blueshift simply increases with *Δ* (Fig. 3c). The result is well reproduced by the calculation (blue solid curve) based on the three-level system model mentioned above. This clarifies that the energy shift in the *σ*^+^*σ*^−^ configuration is only determined by the ATE because of the different transition selection rules (Fig. 3d). Here, we note that although the calculated *δE* in the *σ*^+^*σ*^+^ configuration explains the behavior of the observed energy shift despite the simplicity of the model, it shows a deviation from the experimental results when the pump energy is around 1.6 eV (Fig. 3a). Because the energy spacing between |−1/2 >^v^ and |+3/2>^he^ is estimated to be 3.1 eV, we find that the deviation appears when the pump energy is near the two-photon resonance with the transition between |−1/2 >^v^ and |+3/2>^he^. In addition, such a discrepancy is not discerned in the *σ*^+^*σ*^−^ configuration, where angular momentum conservation forbids the two-photon transition between |+1/2 >^v^ and |+1/2 >^le^. Therefore, we expect that the discrepancy originates from simultaneous excitation of the band-edge and inter-CB transitions, which is not included in our simple three-level model. In fact, the significant effect of two-photon resonance on coherent optical responses has been reported in a three-level ladder-type quantum well system^36^.

## Discussion

**Fig. 4:**
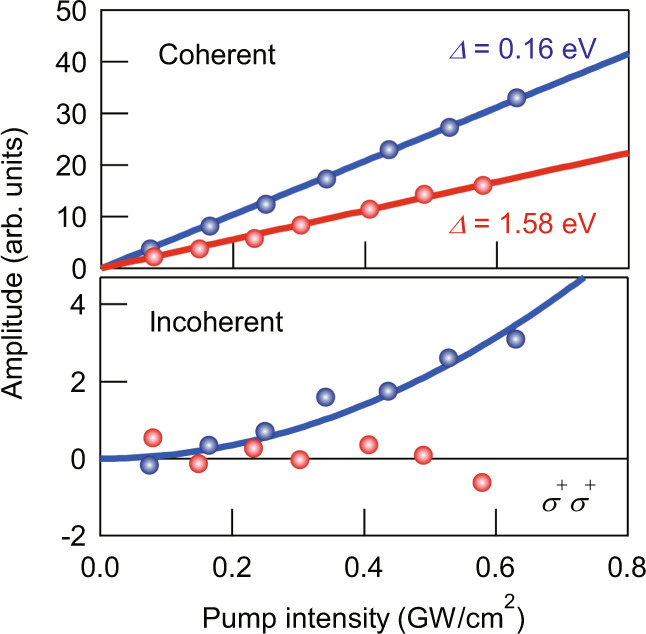
Dependences of the coherent and incoherent components on the detuning energy *Δ*. The amplitudes of coherent and incoherent components estimated from the transient absorption signals in the *σ*^+^*σ*^+^ configuration are shown in the top and bottom panels, respectively. The data are provided as a function of the pump intensity for *Δ* = 0.16 (blue circles) and 1.58 eV (red circles). The solid lines in the top panel describe a linear dependence on the pump intensity and the solid curve in the bottom panel represents a square dependence.

Figure 3a, c shows that the enhancement of the *δE* due to the ATE becomes prominent for $\hslash$*ω* < *E*_0_/2, which lies in the telecommunication wavelength region. For example, in the *σ*^+^*σ*^+^ configuration, the *δE* for *Δ* = 1.58 eV ($\hslash$*ω* = 0.94 eV = 0.37*E*_0_) is comparable to the value of *δE*_Stark_ for *Δ* = 0.3 eV ($\hslash$*ω* = 2.22 eV = 0.88*E*_0_). The large *δE* for excitation energies lower than the two-photon absorption threshold provides a unique opportunity to realize efficient ultrafast optical switching without incoherent effects such as those due to real excitation of excitons or lattice heating, which are detrimental for ultrafast modulation^30,37^. Thus, we investigated the coherent and incoherent contributions to Δ*α* for *Δ* = 0.16 and 1.58 eV. Figure 4 shows the amplitudes of the coherent and incoherent components derived from Δ*α* within the probe energy range from 2.42 to 2.52 eV in the *σ*^+^*σ*^+^ configuration. The amplitudes of both coherent components for *Δ* = 0.16 and 1.58 eV are proportional to *I*_pump_, which corresponds to the *δE* (Supplementary Fig. S7). On the other hand, the amplitude of the incoherent component increases proportionally to *I*_pump_^2^ for *Δ* = 0.16 eV, while no incoherent component is observed for *Δ* = 1.58 eV. This shows that in the case of *Δ* = 0.16 eV ($\hslash$*ω* = 2.36 eV = 0.94*E*_0_), an incoherent component arises from the real excitation of excitons by two-photon absorption, while for *Δ* = 1.58 eV ($\hslash$*ω* < *E*_0_/2), two-photon absorption does not occur. In addition to the suppression of the pump-induced exciton population, near-infrared pulses also do not induce resonant excitations of low-energy modes such as optical phonons in LHPs^38^. Therefore, sizable exciton energy shifts without incoherent background signals and optical damage of the material are expected to occur under higher intensity excitation conditions. Such novel optical manipulation schemes enabled by strong spin–orbit coupling provide a new route to efficient coherent control and modulation.

## Methods

### Sample preparation

We slightly modified the previously reported hot-injection method^22,39^ to synthesize the CsPbBr_3_ NCs with cuboidal shape. All synthetic procedures were performed with typical Schlenk techniques under a dry nitrogen atmosphere. A mixture of Cs_2_CO_3_ (160 mg), oleic acid (0.5 mL), and 1-octadecene (6 mL) was heated at 150 °C for 30 min and then cooled to 100 °C before this Cs-oleate solution was stored. Separately, PbBr_2_ (138 mg), oleic acid (1 mL), oleylamine (1 mL), and 1-octadecene (10 mL) were mixed and degassed in a vacuum at 100 °C for 30 min. After this mixture was heated to 150 °C, 0.8 mL of the Cs-oleate solution was rapidly injected under vigorous stirring. The reaction solution was held at 150 °C for 5 s and then quenched immediately in an ice-water bath to room temperature. Purification and size selection of the obtained polydisperse CsPbBr_3_ NCs were performed by precipitation fractionation with centrifugation in dried *n*-hexane. Transmission electron microscopy observation of resulting NCs characterized their average cuboid edge length to be 6.9 ± 0.7 nm (Supplementary Fig. S1).

### Circularly polarized pump–probe spectroscopy

We divided the output from a regenerative amplifier (with a central wavelength of 1028 nm, a repetition rate of 10 kHz, and a pulse duration of 300 fs) into two beams for the pump- and probe-pulse generation. We used an optical parametric amplifier for the pump-pulse generation. The white-light probe pulse was generated by focusing the beam into water contained in a 10-mm-thick quartz cell. The *σ*^+^/*σ*^−^ probe pulses were obtained using an achromatic quarter-waveplate and the *σ*^+^ pump pulses were generated using a Berek compensator. The chirp of the probe pulse was calibrated by measuring the cross-correlation between the pump and probe pulses. The delay time between pump and probe pulses was controlled by a mechanical delay stage. The CsPbBr_3_ NCs were dispersed in hexane contained in a 1-mm-thick quartz cell and the solution was stirred with a magnetic stirrer during the measurements to avoid the photo-charging of the NCs. The background signal from hexane was removed by subtracting the transient absorption response of a hexane reference sample from the transient absorption data of the CsPbBr_3_ NCs (Supplementary Fig. S2).

## Supplementary information

Supplementary Information

## Data Availability

The data that support the findings of this study are available from the corresponding author upon reasonable request.
