# Supplementary material for: Strong spin-orbit coupling inducing Autler-Townes effect in lead halide perovskite nanocrystals
Source: Nat Commun. 2021 May 21;12:3026. doi: 10.1038/s41467-021-23291-w (PMC8140147; doi:10.1038/s41467-021-23291-w)
Supplement: Supplementary file 1 — Supplementary Information [file 41467_2021_23291_MOESM1_ESM.pdf]

Supplementary Information

Strong spin-orbit coupling inducing Autler-Townes effect in lead halide perovskite nanocrystals

Go Yumoto[1], Hideki Hirori[1], Fumiya Sekiguchi[1], Ryota Sato[1], Masaki Saruyama[1], Toshiharu Teranishi[1] and Yoshihiko Kanemitsu[1]

[1]Institute for Chemical Research, Kyoto University, Uji, Kyoto 611-0011, Japan.

S1. Spectral weight transfer method

We estimated the band-edge exciton transition energy shift δE using the spectral weight transfer (SWT) method[1-4]. We defined the SWT as

$$\mathrm{SWT} = \int_0^{E_0} \Delta\alpha(E)dE \tag{S1}$$

where E_0 is the steady-state band-edge exciton transition energy and $\Delta\alpha(E)$ is the transient absorption spectrum. Because we can write $\Delta\alpha(E)$ as $\Delta\alpha(E) = \alpha(E - \delta E) - \alpha(E) = -\delta E\, d\alpha(E)/dE$, the energy shift δE is given by

$$\delta E = -\frac{\mathrm{SWT}}{\alpha(E_0)} \tag{S2}$$

where we used the condition of $\alpha(0) = 0$.

S2. Estimation of the dipole moment from the optical Stark shift

When a two-level system is driven by an electromagnetic field of $E(t) = E(e^{i\omega t}+e^{-i\omega t})/2$, the excitonic energy shift due to the optical Stark effect (OSE) is given by[1,2]

$$\delta E_{\mathrm{Stark}} = -\Delta+\sqrt{\Delta^2+(\mu E)^2} \tag{S3}$$

with the detuning energy Δ and the transition dipole moment μ. The amplitude of the electromagnetic field E including the local field correction can be described using the local field factor F[3,5]:

$$E^2 = 2|F|^2 I/\left(c\varepsilon_0\sqrt{\varepsilon_{\mathrm{hexane}}}\right)$$
$$F = 3\varepsilon_{\mathrm{hexane}}/(\varepsilon_{\mathrm{NCs}}+2\varepsilon_{\mathrm{hexane}}) \tag{S4}$$

Here I is the intensity of the electromagnetic field, c is the speed of light, ε_0 is the vacuum permittivity, and ε_{hexane} and ε_{NCs} denote the dielectric constants of hexane and $CsPbBr_3$ NCs. We used $\varepsilon_{hexane,}$ = 1.89[6] and ε_{NCs} = 4.96[7], and I was derived using $I = P/(\pi r^2 \Delta t)$, where P is the pulse energy, r is the $1/e^2$ radius, and Δt is the pulse duration. To estimate the transition dipole moment, we fitted the pump-intensity dependence of the energy shift δE using equations (S3) and (S4).

S3. Incoherent components in $\Delta\alpha$ for Δ = 0.16 eV

Figure S3a shows the dynamics of $\Delta\alpha$ probed at 2.55 eV for Δ = 0.16 eV and I_{pump} = 0.56 GW/cm^2. In addition to the coherent component under the pump excitation, the incoherent signal can be discerned after the pump irradiation. To determine the contribution of the incoherent component to the total $\Delta\alpha$, we fitted the signal in $\Delta\alpha$ dynamics at every probe energy to the following phenomenological equation:

$$\Delta\alpha(t) = A_1[1+\mathrm{erf}(t/\Delta t)]-A_2\exp(-t/\tau)\times[1+\mathrm{erf}(t/\Delta t-\Delta t/2\tau)] \quad \text{(S5)}$$

Here τ represents the phenomenological spin-flip time, A_1 denotes the amplitude of the exciton with J_{ex} = +1, which is assumed to exhibit no decay in our time window, and A_2 is the amplitude of the state transfer to the exciton with J_{ex} = −1[8]. The fitting result (Fig. S3a: black curve) shows a good agreement with the data. By subtracting the derived incoherent components from the total $\Delta\alpha$, we obtained the coherent components. Figure S3b shows the data at the probe energy of 2.55 eV that were obtained by this subtraction procedure.

S4. Determination of the inter-CB transition energy

We performed pump-probe experiments under resonant excitation of the band-edge exciton transitions and measured the pump-induced change in the absorption spectra by changing the energy of the monochromatic probe pulses in the ultraviolet to visible range, which were generated from an optical parametric amplifier. In this experiment, we used a regenerative amplified mode-locked Ti:sapphire laser system with a repetition rate of 1 kHz equipped with two optical parametric amplifiers for generating linearly polarized pump and probe pulses. The pump pulse with a photon energy of 2.53 eV and fluence of 17 μJ/cm^2 resonantly excites the samples. The pump-induced change in the probe pulse was measured using a balanced detection technique. The pump beam was modulated at a frequency of 200 Hz by an optical chopper. To clearly detect the pump-induced absorption change in the UV range, where the optical density of the samples is higher than that in the visible range, we used samples that were diluted to have about half of the optical density of the samples used in the main manuscript.

Figure S5a shows the pump-probe delay dependence of the pump-induced change in the absorption spectra $\Delta\alpha$ probed at 2.84, 3.10 and 3.22 eV. While a positive $\Delta\alpha$, corresponding to induced absorption, is observed for all probe energies, we can see that the amplitude of the induced

absorption is smaller for a probe energy of 3.10 eV, where there is an additional absorption band in the steady-state absorption spectrum (Fig. S5b). Because the signals shown in Fig. S5a do not decay within the observed time window, we averaged the signals in the range of the pump-probe delay later than 1 ps to obtain the spectral information on $\Delta\alpha$. The resulting $\Delta\alpha$ spectrum is shown in Fig. S5c, and a clear dip can be discerned in the induced absorption spectrum around a probe energy of 3.1 eV, corresponding to absorption bleaching. Whereas the induced absorption observed in the whole probe energy range can be attributed to the pump-induced refractive index change[9], the dip structure shows the presence of a transition which causes absorption bleaching under resonant excitation. An additional absorption band which shows bleaching under resonant excitation is reported to be ascribed to transitions between the valence band and the heavy and light electron CB states[10]. Therefore, we find that the energy spacing Δ_{so} of the inter-CB transitions is 0.58 eV. This value is consistent with the experimentally reported value of the spin-orbit splitting energy of 0.8 eV in $MAPbBr_3$ single crystals[11]. Note that size confinement can be considered to have little effect on Δ_{so} because the size-confinement-induced change in the energy spacing between spin-orbit split states is estimated to be 0.03 eV (see Supplementary Text S5). Thus, the slightly smaller value in nanocrystals compared to 0.8 eV presumably stems from the reduced spin-orbit coupling due to structural distortions[12].

S5. Estimation of confinement energy

The confinement energy for halide perovskite NCs can be described by the expression $(\hbar\pi)^2/(2m^*L^2)$, where L is the side length of the NC, and m^* is the reduced mass of the exciton[7,13,14]. By using this relation and m^* =0.132 m_0 (m_0: the free electron mass)[15], we estimated the confinement energy of our samples to be 0.06 eV. Because the electron and hole effective masses are similar in $CsPbBr_3$[7], the size confinement effect is expected to raise the band-edge conduction band states by the half of the confinement energy[13]. Therefore, the confinement-induced change in the energy spacing between spin-orbit split states is 0.03 eV, which is much smaller than the Δ_{so} of 0.58 eV.

S6. Estimation of μ' from the pump-intensity dependence of the residual energy shift

To estimate the values of the transition dipole moment μ' of the inter-CB transitions, we defined the difference between the observed energy shift δE and δE_{Stark} as the residual energy shift $\delta E_{residual}$. For the $\sigma^+\sigma^+$ configuration, we used the δE_{Stark} calculated from equation (1) in the main manuscript with μ = 19 D. For the $\sigma^+\sigma^-$ configuration, we used δE_{Stark} = 0. The pump-intensity dependences of $\delta E_{residual}$ for Δ = 1.26 and 1.58 eV are shown in Fig. S6. We find that the $\delta E_{residual}$ is independent of the probe polarization. In addition, it is seen that the $\delta E_{residual}$ is larger for Δ = 1.58 eV. This indicates that as the pump energy is decreased, the excitation condition approaches a transition resonance and the pump-induced change of the transition can be probed by both the probe polarizations. Therefore these behaviors indicate that the inter-CB transitions contribute to the energy shift in the Autler-Townes effect region. We assumed that the residual energy shift can be described by

$$\delta E_{\rm residual} = 0.5\times\left((-\Delta') + \sqrt{(-\Delta')^2 + (\mu' E)^2}\right) \quad \text{(S6)}$$

and performed a global fitting of the data shown in Fig. S6 with $\Delta_{\rm so}$ fixed to 0.58 eV and μ' being only the shared fitting parameter. Equation (S6) corresponds to the expression of the upper ground-state band shift in the two-level OSE[16]. Here $\Delta' = \hbar\omega - \Delta_{\rm so}$ and the factor 0.5 in equation (S6) reflects the fact that we only consider the energy shift of the conduction band-edge state in the Autler-Townes effect. The fitting results (black lines in Fig. S6) well reproduce the experimental results and we obtained μ' = 25 D.

S7. Calculation of energy shift in the three-level system

To calculate the observed energy shift δE, in accordance with the transition selection rules, we assumed that the three-level systems consist of $|-1/2>^{\rm v}$, $|+1/2>^{\rm so}$, and $|+3/2>^{\rm he}$ for the $\sigma^+\sigma^+$ configuration, and $|+1/2>^{\rm v}$, $|-1/2>^{\rm so}$, and $|+1/2>^{\rm le}$ for the $\sigma^+\sigma^-$ configuration. We modeled each system by using an effective Hamiltonian (H_+ for the $\sigma^+\sigma^+$ configuration and H_- for the $\sigma^+\sigma^-$ configuration) with the rotating-wave approximation.

$$\begin{aligned} H_+ = {} & \varepsilon_{\rm v}|-1/2>^{\rm v}<-1/2|^{\rm v} + \varepsilon_{\rm so}|+1/2>^{\rm so}<+1/2|^{\rm so} + \varepsilon_{\rm he}|+3/2>^{\rm he}<+3/2|^{\rm he} + \hbar\omega a^\dagger a \\ & + (\mu E/2) \times [|-1/2>^{\rm v}<+1/2|^{\rm so}a^\dagger + |+1/2>^{\rm so}<-1/2|^{\rm v}a] \\ & + (\mu' E/2) \times [|+1/2>^{\rm so}<+3/2|^{\rm he}a^\dagger + |+3/2>^{\rm he}<+1/2|^{\rm so}a] \end{aligned}$$

$$\begin{aligned} H_- = {} & \varepsilon_{\rm v}|+1/2>^{\rm v}<+1/2|^{\rm v} + \varepsilon_{\rm so}|-1/2>^{\rm so}<-1/2|^{\rm so} + \varepsilon_{\rm le}|+1/2>^{\rm le}<+1/2|^{\rm le} + \hbar\omega a^\dagger a \\ & + (\mu' E/2) \times [|-1/2>^{\rm so}<+1/2|^{\rm le}a^\dagger + |+1/2>^{\rm le}<-1/2|^{\rm so}a] \end{aligned} \quad \text{(S7)}$$

Here $\varepsilon_{\rm i}$ represents the energy of a state with index i (i = v, so, he, or le), and $a^\dagger$ (a) is the photon creation (annihilation) operator. Furthermore, we assume $\varepsilon_{\rm le} = \varepsilon_{\rm he}$. In H_- there is no term that connects $|+1/2>^{\rm v}$ and $|-1/2>^{\rm so}$ because this transition is not accessible via the σ^+ pump light. Since $|+1/2>^{\rm so}$ couples to the photon-dressed states $|-1/2 + \hbar\omega>^{\rm v}$ and $|+3/2 - \hbar\omega>^{\rm he}$ in the $\sigma^+\sigma^+$ configuration, we can write the matrix of H_+ for the basis of $|+1/2>^{\rm so}$, $|-1/2 + \hbar\omega>^{\rm v}$, and $|+3/2 - \hbar\omega>^{\rm he}$ as

$$\begin{pmatrix} \varepsilon_{\rm so} & \mu E/2 & \mu' E/2 \\ \mu E/2 & \varepsilon_{\rm v} + \hbar\omega & 0 \\ \mu' E/2 & 0 & \varepsilon_{\rm he} - \hbar\omega \end{pmatrix} \quad \text{(S8)}$$

The eigenenergy and the energy shift of $|+1/2>^{\rm so}$ can be estimated by diagonalizing this matrix. The same procedure can be used to estimate the energy shift of $|-1/2>^{\rm v}$, which only couples to the photon-dressed state $|+1/2 - \hbar\omega>^{\rm so}$. From the energy shifts of $|+1/2>^{\rm so}$ and $|-1/2>^{\rm v}$, we obtain δE for the $\sigma^+\sigma^+$ configuration. The δE for the $\sigma^+\sigma^-$ configuration can be derived by performing a similar calculation.

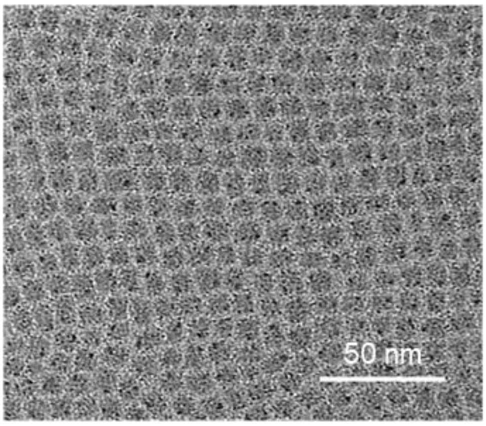

Fig. S1 | Transmission electron microscopy image. Transmission electron microscopy (TEM) image of $CsPbBr_3$ NCs.

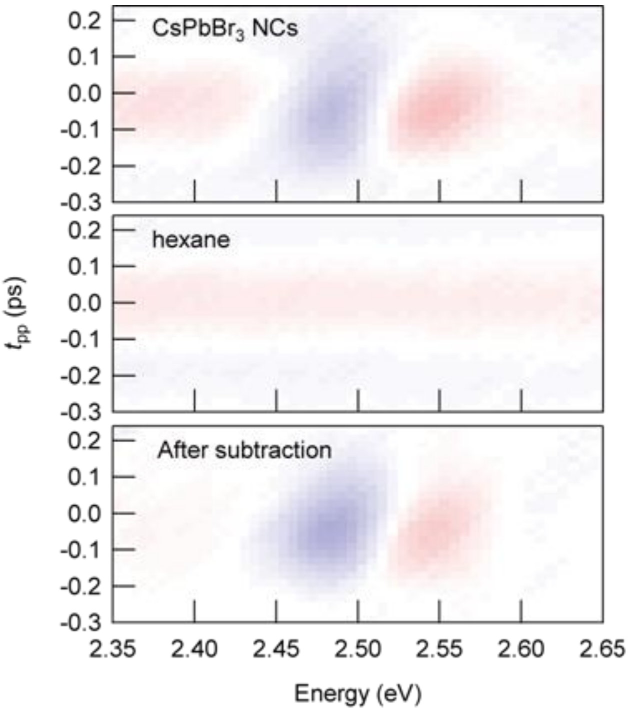

Fig. S2 | Subtraction of background signal from hexane. Transient absorption signals from $CsPbBr_3$ NCs (top panel) and a hexane reference sample (middle panel) for Δ = 1.58 eV. The bottom panel shows the data after removal of the background signal from hexane.

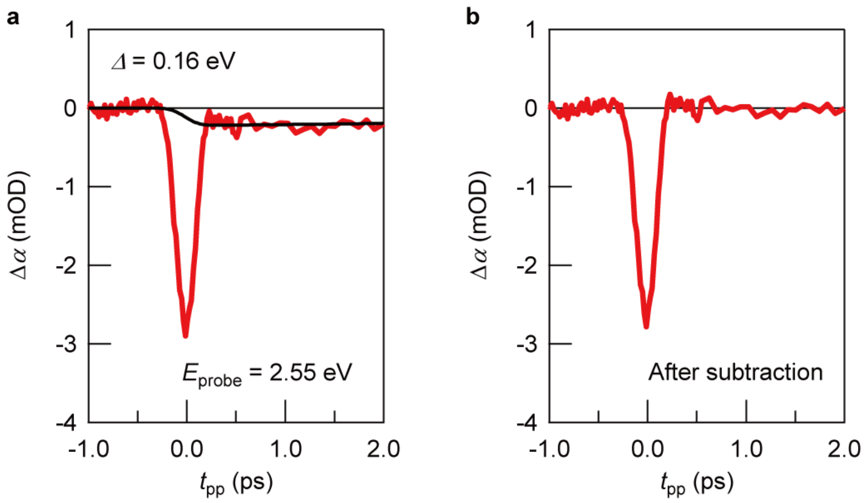

Fig. S3 | Coherent and incoherent components for $\mathit{\Delta}$ = 0.16 eV. a, Raw data of the dynamics of $\Delta\alpha$ probed at 2.55 eV for $\mathit{\Delta}$ = 0.16 eV. The pump intensity was 0.56 GW/cm^2. The black curve is the fitting result of the incoherent component. **b**, Coherent component of the $\Delta\alpha$ dynamics shown in **a**.

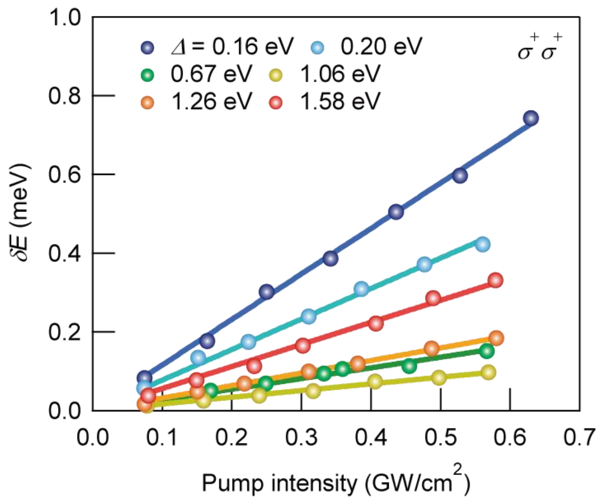

Fig. S4 | Pump-intensity dependence of the energy shift in the $\sigma^+\sigma^+$ configuration. Energy shift as a function of pump intensity for different detuning energies Δ in the $\sigma^+\sigma^+$ configuration. The lines are linear fitting results.

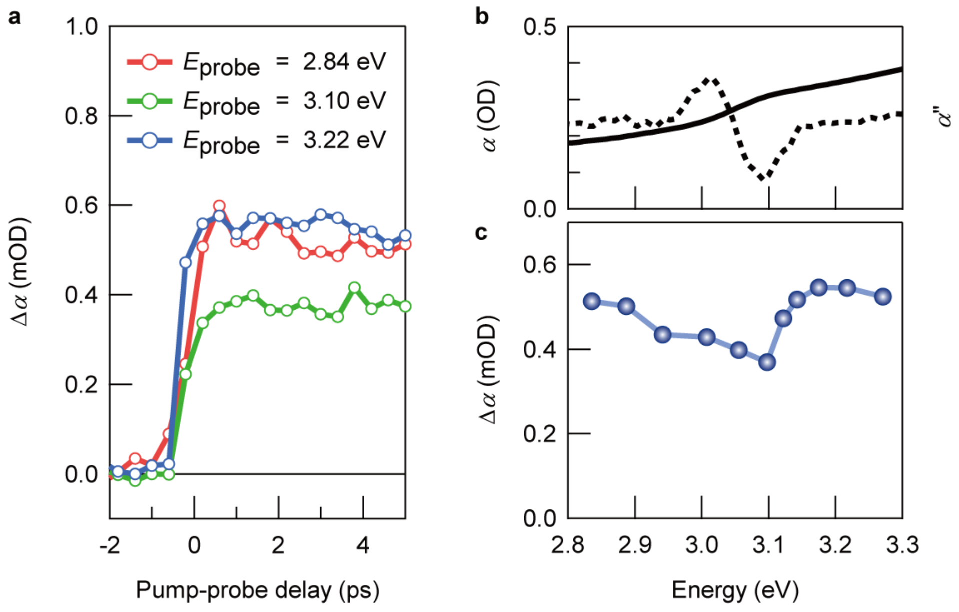

Fig. S5 | Pump-induced absorption change under resonant excitation of the band-edge exciton transitions. **a**, Transient absorption dynamics measured by probe pulses with photon energies of 2.84, 3.10 and 3.22 eV. **b**, Absorption spectrum (solid curve, left axis) and its second derivative (dashed curve, right axis). **c**, Pump-induced change in the absorption spectrum under resonant excitation.

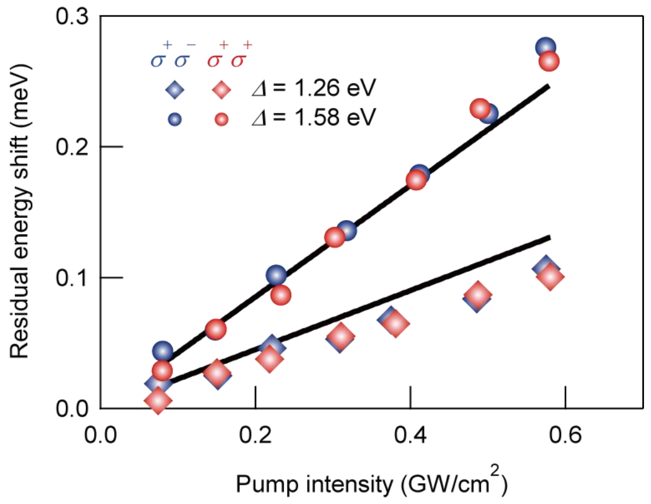

Fig. S6 | Pump-intensity dependence of the residual energy shift. The residual energy shifts for Δ = 1.26 and 1.58 eV are shown with the diamonds and circles, respectively. Data for the $\sigma^+\sigma^-$ and the $\sigma^+\sigma^+$ configurations are shown in blue and red, respectively. The black lines are fitting results.

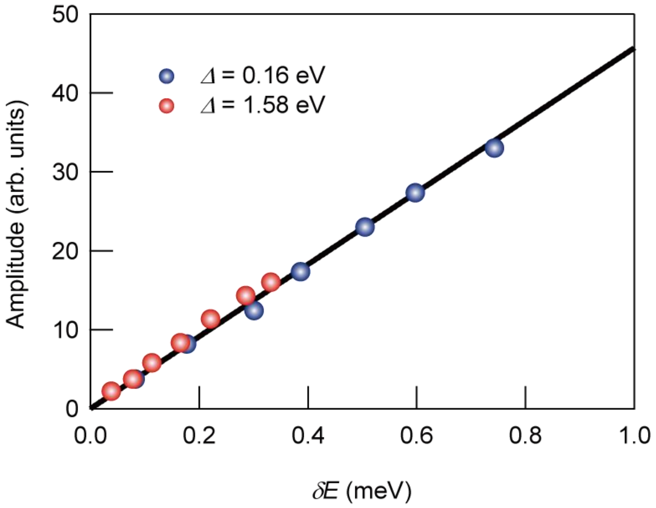

Fig. S7 | Amplitude of the coherent component as a function of the energy shift. Amplitudes of coherent components as a function of energy shift for Δ = 0.16 (blue circles) and 1.58 (red circles) eV in the $\sigma^+\sigma^+$ configuration. We fitted both data sets to a single linear function (black line).

References

1. Sie, E. J. *et al*. Valley-selective optical Stark effect in monolayer WS_2. *Nat. Mater.* **14**, 290-294 (2015).
2. Giovanni, D. *et al*. Tunable room-temperature spin-selective optical Stark effect in solution-processed layered halide perovskites. *Sci. Adv.* **2**, e1600477 (2016).
3. Li, Y., He, S., Luo, X., Lu, X. & Wu, K. Strong spin-selective optical Stark effect in lead halide perovskite quantum dots. *J. Phys. Chem. Lett.* **11**, 3594-3600 (2020).
4. Proppe, A. H. *et al*. Transition dipole moments of n = 1, 2, and 3 perovskite quantum wells from the optical Stark effect and many-body perturbation theory. *J. Phys. Chem. Lett.* **11**, 716-723 (2020).
5. Makarov, N. S. *et al*. Spectral and dynamical properties of single excitons, biexcitons, and trions in cesium–lead-halide perovskite quantum dots. *Nano Lett.* **16**, 2349-2362 (2016).
6. Raikar, U. S. *et al*. Solvent effects on the absorption and fluorescence spectra of coumarins 6 and 7 molecules: Determination of ground and excited state dipole moment. *Spectrochim. Acta A* **65**, 673-677 (2006).
7. Protesescu, L. *et al*. Nanocrystals of cesium lead halide perovskites ($CsPbX_3$, X = Cl, Br, and I): novel optoelectronic materials showing bright emission with wide color gamut. *Nano Lett.* **15**, 3692-3696 (2015).
8. Giovanni, D. *et al*. Highly spin-polarized carrier dynamics and ultralarge photoinduced magnetization in $CH_3NH_3PbI_3$ perovskite thin films. *Nano Lett.* **15**, 1553-1558 (2015).
9. Price, M. B. *et al*. Hot-carrier cooling and photoinduced refractive index changes in organic–inorganic lead halide perovskites. *Nat. Commun.* **6**, 8420 (2015).
10. Leguy, A. M. A. *et al*. Experimental and theoretical optical properties of methylammonium lead halide perovskites. *Nanoscale* **8**, 6317-6327 (2016).
11. Ohara, K. *et al*. Impact of spin-orbit splitting on two-photon absorption spectra in a halide perovskite single crystal. *Phys. Rev. B* **103**, L041201 (2021).
12. McKechnie, S. *et al*. Dynamic symmetry breaking and spin splitting in metal halide perovskites. *Phys. Rev. B* **98**, 085108 (2018).
13. Schmitt-Rink, S., Miller, D. A. B. & Chemla, D. S. Theory of the linear and nonlinear optical properties of semiconductor microcrystallites. *Phys. Rev. B* **35**, 8113-8125 (1987).
14. Yumoto, G. *et al*. Hot biexciton effect on optical gain in $CsPbI_3$ perovskite nanocrystals. *J. Phys. Chem. Lett.* **9**, 2222-2228 (2018).
15. Sercel, P. C., Lyons, J. L., Bernstein, N. & Efros, A. L. Quasicubic model for metal halide perovskite nanocrystals. *J. Chem. Phys.* **151**, 234106 (2019).
16. Cunningham, P. D., Hanbicki, A. T., Reinecke, T. L., McCreary, K. M. & Jonker, B. T. Resonant optical Stark effect in monolayer WS_2. *Nat. Commun.* **10**, 5539 (2019).
